# Transcatheter Mitral Valve Implantation (TMVI) Using Edwards SAPIEN 3 Prostheses in Patients at Very High or Prohibitive Surgical Risk: A Single-Center Experience

**DOI:** 10.1155/2020/9485247

**Published:** 2020-01-06

**Authors:** Nicolas Werner, Caroline Kilkowski, Dorothee Sutor, Udo Weisse, Steffen Schneider, Ralf Zahn

**Affiliations:** ^1^Medizinische Klinik B, Klinikum Ludwigshafen, Ludwigshafen, Germany; ^2^Klinik für Herzchirurgie, Klinikum Ludwigshafen, Ludwigshafen, Germany; ^3^Institut für Herzinfarktforschung, Ludwigshafen, Germany

## Abstract

**Background:**

Mitral valve surgery in patients with failing bioprosthesis, annuloplasty rings, or in patients with advanced mitral annular calcification (MAC) is associated with high morbidity and mortality rates. Percutaneous antegrade transseptal transcatheter mitral valve implantation (TMVI) has recently successfully been performed in those patients at high or prohibitive surgical risk, but data on patients treated by TMVI are sparse. This study sought to evaluate short- and midterm outcomes of patients treated by TMVI at our site in clinical practice.

**Methods and Results:**

From October 2016 to February 2018, seven patients (six women and one man) at high or prohibitive surgical risk underwent TMVI at our site. Three procedures were performed as TMVI in failed mitral valve bioprostheses (TMVI-VIV, “valve-in-valve”), one procedure was performed as TMVI in a failed mitral annuloplasty ring (TMVI-R), and three procedures were performed as TMVI in advanced native mitral annular calcification (TMVI-MAC). Mean age of the population treated was 77 ± 8.1 years, and mean log EuroScore I was 39 ± 0.12%. In all patients, an Edwards SAPIEN 3 transcatheter heart valve was implanted under 3D-TOE and fluoroscopic guidance using a transvenous/transseptal access. Indication for TMVI was the presence of advanced heart failure symptoms in all patients (NYHA class III/IV). The predominant dysfunction of the mitral valve treated was severe regurgitation in 72% (*n* = 5) and severe stenosis in 29% (*n* = 2) of all patients. TMVI was technically successful in all procedures. Clinical success with functional improvement of at least one NYHA class after procedure compared with before procedure was also achieved in all patients. Median NYHA class improved significantly from 4 before procedure to 2 after TMVI (*p*=0.008). Mitral valve regurgitation was reduced to trace or mild in all but one patient, who showed moderate MR after TMVI-MAC. No patient-prosthesis mismatch or LVOT obstruction occurred after TMVI. Two patients underwent interventional ASD closure during the in-hospital course due to a large and persisting atrial septal defect after transseptal access. One patient underwent pacemaker implantation due to complete AV-block after TMVI. One patient died in hospital 12 days after the procedure due to severe hospital-acquired pneumonia and sepsis. In-hospital mortality rate was 14% (1/7) in this high-risk population. After hospital discharge, no death occurred and clinical improvement—according to NYHA functional class—remained stable during one-year follow-up.

**Conclusion:**

In this small single-center series, TMVI appears promising for patients at high or prohibitive surgical risk with either failing mitral bioprostheses/annuloplasty rings or native mitral valve dysfunction in combination with advanced MAC. Gaining experience in TMVI and new valves will further improve safety and efficacy of this new treatment option.

## 1. Introduction

Mitral valve surgery is presently the recommended first-line treatment option for the majority of patients with severe mitral valve regurgitation or stenosis, who are at an acceptable surgical risk. However, reoperation is needed in approximately one-third of patients after mitral valve replacement or repair during 10-year follow-up [1]. In addition, a relevant proportion of implanted mitral bioprosthesis degenerate over time and need further treatment due to clinically relevant prosthetic stenosis or regurgitation [2]. Redo mitral valve surgery is associated with high mortality, especially in elderly patients with high comorbidities at high surgical risk [3, 4]. Over the past years, transcatheter aortic valve replacement (TAVR) has been established as a safe treatment option for degenerated aortic valve bioprosthesis (valve-in-valve), especially in patients at an increased risk for redo surgery [5]. Recently, transcatheter mitral valve implantation (TMVI) has successfully been performed as a treatment for degenerated mitral bioprosthesis (transcatheter mitral valve-in-valve implantation, TMVI-VIV) or failed mitral annuloplasty rings (transcatheter mitral valve-in-ring implantation, TMVI-R) in patients at very high or prohibitive surgical risk [6–8].

Patients with severe symptomatic mitral valve dysfunction and advanced mitral annular calcification (MAC) are a special, usually elderly and frail, subpopulation at an increased surgical risk due to relevant comorbidities [9, 10]. In those patients, surgical mitral valve replacement carries higher procedural mortality and is associated with suboptimal technical results due to the high calcium burden of the mitral ring [11]. Recently, TMVI into the calcified native mitral valve has successfully been performed using a balloon-expandable transcatheter heart valve in patients with MAC (TMVI-MAC) [12–17]. Although those first technical and clinical results of these complex procedures are encouraging, information on mid- or long-term safety and efficacy of TMVI is generally sparse to date.

In the present study, we sought to evaluate the short- and midterm clinical outcomes of patients treated by TMVI (performed as TMVI-VIV, TMVI-R, or TMVI-MAC) at our site.

## 2. Methods

### 2.1. Patient Population

We performed a single-center registry from the beginning of our TMVI program in October 2016. Each patient underwent complete transthoracic (TTE) and 3D-transesophageal echocardiography (TOE) preprocedurally. All procedures were performed using either an iE33 or an Epiq 7 ultrasound system (Philips Medical System, Andover, Massachusetts) with a fully sampled 3D matrix array TOE transducer. Mitral valve or prosthetic regurgitation was evaluated in TTE/TOE and angiography before procedure; severity of mitral regurgitation was classified as no regurgitation, mild, moderate, or severe according to an integration of established parameters like vena contracta, jet area, jet density, jet deceleration rate, and systolic pulmonary venous flow reversal [18]. Mitral valve or prosthetic stenosis was determined by TTE/TOE using established parameters, like mean pressure gradient (pmean) in continuous- or pulsed-wave Doppler, deceleration rate, and 3D-planimetry in TOE. Severity of stenosis was classified as no stenosis, mild, moderate, or severe according to echocardiographic and invasive measurements. All patients underwent complete diagnostic left and right heart catheterization before TMVI. In all patients, no signs of infective endocarditis were present in blood samples, blood cultures, and TOE before TMVI. Relevant paravalvular regurgitation was excluded in all patients with prior surgical mitral valve replacement by 3D-TOE prior to TMVI.

In patients without an indication for long-term oral anticoagulation, dual antiplatelet therapy (100 mg aspirin and 75 mg clopidogrel) was given for 3 months after TMVI. In patients with an indication for permanent oral anticoagulation, 75 mg clopidogrel was added for four weeks after TMVI. All naive patients received either a loading dose of 500 mg of aspirin and 600 mg of clopidogrel (no oral anticoagulation) or a single loading with 600 mg clopidogrel (in patients with permanent oral anticoagulation) on the day prior to procedure.

Risk of open surgical repair was calculated for each patient using the Log. EuroSCORE I. Each patient was discussed in the local Heart Team prior to procedure, and in every patient, TMVI was favored instead of reoperation by the Heart Team. All available therapeutical options and procedural risk of TMVI were discussed with each patient, and written informed consent was obtained. Each patient was informed about the off-label use of the implanted Edwards SAPIEN 3 transcatheter heart valve in mitral position prior to procedure.

### 2.2. TMVI Techniques and Procedure

All procedures were performed under general anesthesia by an experienced multidisciplinary team consisting of an interventional cardiologist, echocardiologist, and cardiac anesthesiologist in a hybrid operating room. TMVI was guided by real-time 3D-TOE and fluoroscopy. Antibiotic single-shot prophylaxis (2 g of cefazoline) was administered intravenously prior to procedure. During all procedures, unfractionated heparin was administered to maintain an activated clotting time above 250 seconds. All patients in the underlying population were treated by implantation of the Edwards SAPIEN 3 prosthesis (Edwards Lifesciences, Irvine, CA, USA). At the beginning of the procedure, a temporary pacemaker was placed in the right ventricle to perform “rapid pacing” with the induction of slow flow through the mitral valve during implantation of the transcatheter heart valve. All procedures were performed using an antegrade transseptal approach. Transseptal puncture was performed under TOE guidance in a superior and posterior localization (similar to the position used during MitraClip® implantation) and in the standard technique, as previously described by Brockenbrough and Braunwald [19]. After transseptal puncture, the native mitral valve, bioprosthesis, or annuloplasty ring was crossed with hydrophilic guidewire or a standard 0.035-inch J-guidewire over a steerable guiding catheter (e.g., Agilis, St. Jude Medical, USA). Afterwards, the standard wire was exchanged for an extrasupport wire with its end manually bended as a J-curve placed in the left ventricular apex (e.g., Amplatz Super Stiff Wire) over a standard 5F multipurpose or a pigtail catheter. Prior to transseptal insertion of the Edwards Commander delivery system (Edwards Lifesciences, Irvine, CA, USA) of the transcatheter heart valve into the left atrium, a balloon dilatation of the interatrial septum was performed using an Osypka VACS II 12 to 14 mm Balloon (Osypka AG, Rheinfelden, Germany). No balloon valvuloplasty of the stenosed native mitral valve or bioprosthesis was performed in this series of patients prior to TMVI. Afterwards, the delivery system with the mounted Edwards SAPIEN 3 prosthesis (mounted in the opposite direction as performed for transfemoral TAVI) was carefully inserted into the left atrium and into the mitral valve under maximal flexion of the delivery system. Then, the Edwards SAPIEN 3 prosthesis was delivered into the calcified mitral valve, mitral bioprosthesis, or mitral annuloplasty ring under rapid ventricular pacing (160–180 beats/min). The aim was to reach a position of the prosthesis with its outer skirt exactly placed into the plane of the calcified mitral annulus, into the valvular plane of the bioprosthesis, or into the annuloplasty ring. This was achieved by a slight protrusion of approximately 10–20% of the prosthesis into the left atrium.

Afterwards, an adequate anchoring of the prosthesis was confirmed by fluoroscopy, 2D-TOE, and 3D-TOE. Reduction in mitral valve regurgitation and/or mitral valve stenosis was evaluated by periprocedural TOE. In the underlying series, no additional postdilatation was performed after TMVI. After removal of the delivery system, the size and hemodynamic effect of an iatrogenic atrial septum defect (ASD) was evaluated by TOE. In case of a large ASD after TMVI, TOE follow-up was performed during the days after procedure. For the possibility of spontaneous decrease in the size of the iatrogenic ASD, which is often observed during the days after procedure, interventional ASD closure was not routinely performed immediately after TMVI. Femoral venous access was closed by a standard z-suture followed by manual compression.

Complete technical procedural success was defined as the ability of the device to be deployed as intended and the delivery system successfully retrieved without procedural mortality or the need for emergency surgery or intervention measured at the time of the patient's exit from the cardiac catheterization laboratory (according to the published MVARC criteria [20]). Clinical success was defined as an improvement in at least one NYHA functional class after TMVI measured during in-hospital follow-up compared with prior to procedure. Periprocedural death was defined as any death occurring within 24 hours after the procedure. Complications were assessed according to the MVARC criteria [20]. Routine TTE was performed on the first day after procedure and was repeated before hospital discharge. A patient-prosthesis mismatch was defined as a mean pressure gradient of >5 mmHg after implantation in pulsed-wave Doppler measurement. LVOT obstruction was defined as a newly observed flow maximum of >150 cm/sek in pulsed-waved Doppler measurement after TMVI.

### 2.3. Technique Used for TMVI-MAC

**Figure 1:**
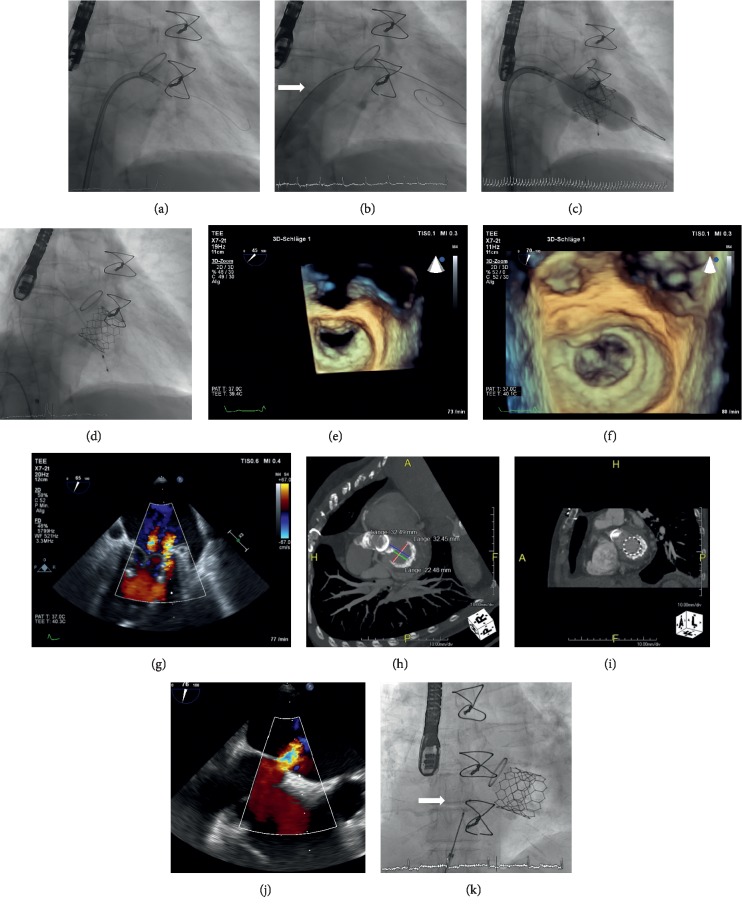
TMVI-MAC: (a) fluoroscopy shows a 0.035-inch J-guide inserted through the mitral valve in the left ventricle via a steerable guiding catheter (Agilis, 8,5F, Abbott, Illinois, USA). (b) Fluoroscopy shows balloon dilatation (Osypka VACS II 12 × 60 mm Balloon, Osypka AG, Germany, white arrow) of the interatrial septum over an Amplatz Super Stiff guidewire (Boston Scientific) to create an artificial atrial septum defect prior to insertion of the prosthesis into the left atrium. (c) *Fluoroscopy* shows the implantation of a 29 mm Edwards SAPIEN 3 (ES 3) prosthesis into the heavily calcified native mitral valve. (d) *Fluoroscopy* shows the final result after implantation of the ES 3 prosthesis into the calcified native mitral valve. (e) 3D-*TEE* shows the native mitral valve with a heavily calcified native mitral ring prior to TMVI (view from left atrium). (f) 3D-TEE shows the result after TMVI of the 29 mm ES 3 prosthesis into the advanced mitral annular calcification (MAC). (g) 2D-*TEE* with color duplex shows only minimal paravalvular and valvular regurgitation after implantation of the ES 3 Prosthesis into the MAC. (h) Cardiac CT-scan shows the exact dimensions of the calcified mitral valve prior to implantation. (i) Cardiac CT-scan shows the final result after TMVI into MAC. (j) 2D-TEE with color duplex shows a large iatrogenic atrial septal defect (ASD) with clinically relevant right-to-left cardiac shunt. (k) *Fluoroscopy* shows successful transcatheter implantation of an Amplatzer Septal Occluder (Abbott, Illinois, USA) into the ASD (white arrow).

In every patient undergoing prosthetic valve-in-mitral-annular-calcification implantation, 3D evaluation of the native mitral annulus was performed by computed tomography (CT) prior to procedure for the determination of the exact dimensions of the calcified native mitral ring and for the confirmation of a continuous calcification of >50% of the circumference of the mitral valve to facilitate a stable anchorage of the implanted transcatheter heart valve (Figure 1). Afterwards, an optimally sized Edwards SAPIEN 3 prosthesis was determined by computer simulation using a 3D-CT-model (3mensio, Pie Medical, Maastricht, the Netherlands). A prosthesis showing an approximately 10% oversizing according to the individual calcified native mitral ring was considered as optimally sized. TMVI was performed as previously described (see Section 2.2).

### 2.4. Technique Used for TMVI-VIV and TMVI-R

**Figure 2:**
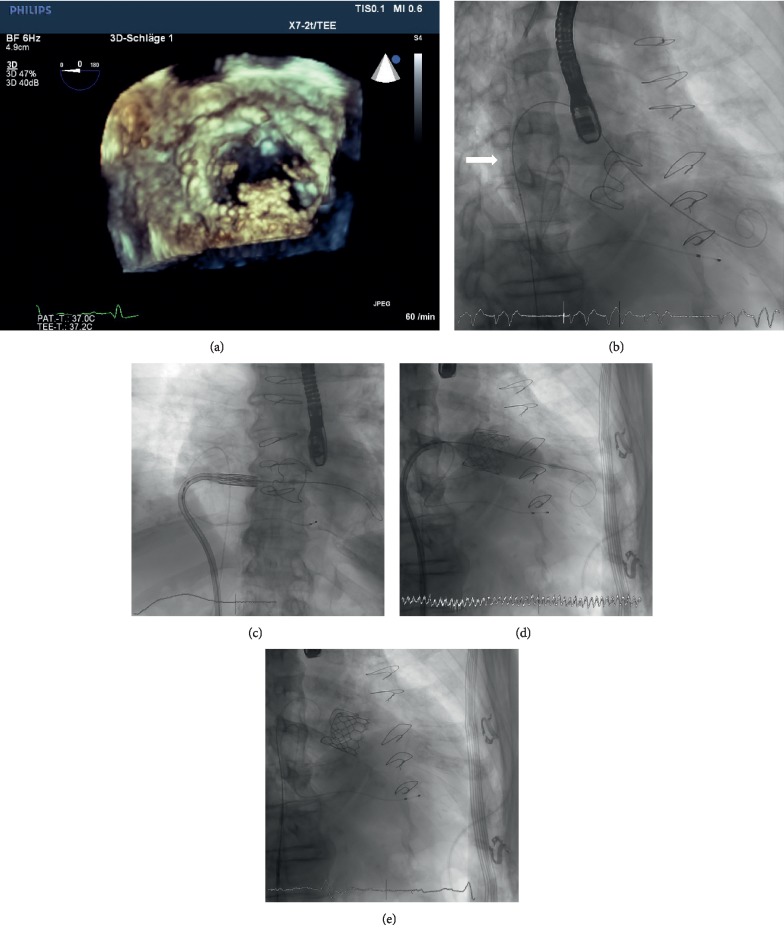
TMVI-VIV: (a) *3D*-TEE shows degenerated biological mitral valve prosthesis (Carpentier Edwards 31 mm biological mitral valve prosthesis) with calcified and thickened leaflets (view from left atrium). (b) Fluoroscopy shows balloon dilatation (Osypka VACS II 12 × 40 mm Balloon, Osypka AG, Germany; white arrow) of the interatrial septum to create an artificial atrial septum defect prior to insertion of the prosthesis into the left atrium. (c) *Fluoroscopy* shows the ES 3 prosthesis after insertion into the left atrium. (d) *Fluoroscopy* shows the implantation of the ES 3 prosthesis into the malfunctioning biological mitral valve prosthesis. (e) *Fluoroscopy* shows the final result of the mitral valve-in-valve implantation.

**Figure 3:**
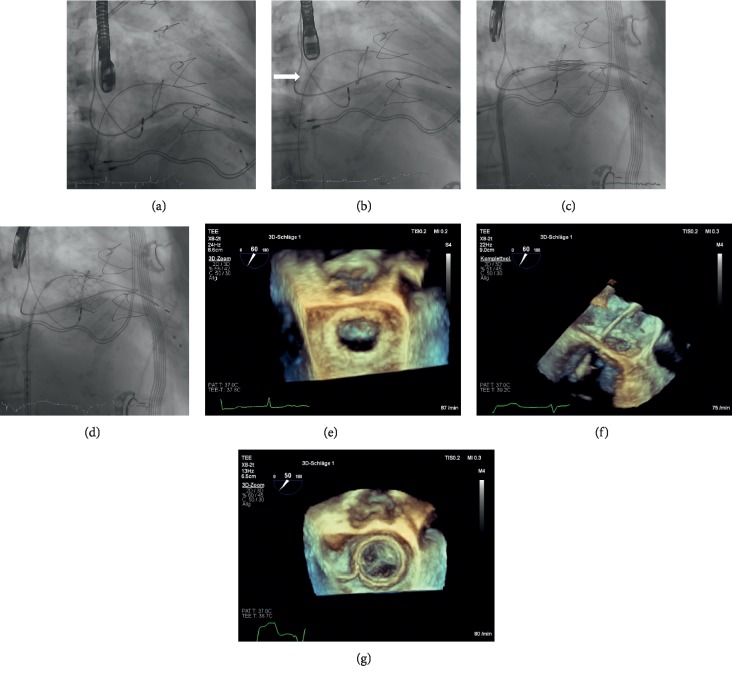
TMVI-R: (a) fluoroscopy shows an Amplatz Super Stiff guidewire (Boston Scientific) inserted in the left ventricle through the 29 mm Duran Annuloplasty Ring after transseptal puncture. (b) Fluoroscopy shows balloon dilatation (Osypka VACS II 14 × 30 mm Balloon, Osypka AG, *Germany*; white arrow) of the interatrial septum to create an artificial atrial septum defect prior to insertion of the prosthesis into the left atrium. (c) *Fluoroscopy* shows the ES 3 prosthesis after insertion into the Duran Annuloplasty Ring prior to implantation. (d) *Fluoroscopy* shows the final result after implantation of the ES 3 prosthesis into the 29 mm Duran Annuloplasty Ring. (e) 3D-TEE shows the previously reconstructed mitral valve by 29 mm Duran Annuloplasty Ring prior to TMVI (view from left atrium). (f) 3D-*TEE* shows the insertion of the delivery catheter through the reconstructed mitral valve prior to implantation (view from lateral). (g) 3D-*TEE* showing the final result after implantation of the 29 mm ES 3 Prosthesis into the Duran Annuloplasty Ring (view from left atrium).

TMVI was performed as previously described (Section 2.2) (Figures 2 and 3). In patients undergoing TMVI-VIV and TMVI-R, the size of the Edwards SAPIEN 3 prosthetic valve was selected according to an integrative approach taking into account the internal dimensions as reported by manufacturers, CT, and TOE, measurements. In each patient, the valve-in-valve app was additionally used for sizing of the transcatheter heart valve prior to procedure (http://www.ubqo.com/vivmitral) [21]. Methods and statistics were performed following one of our previous publications [22].

## 3. Statistics

All patients were prospectively enrolled in our single-center TMVI registry. Data are presented as sample number with percentages in parenthesis unless otherwise indicated. Categorical variables are presented by absolute numbers and percentages. Continuous variables are expressed as means ± standard deviation (SD) or median with 25% and 75% quartile (interquartile range, IQR). Changes in NYHA class were statistically assessed using sign test. *p* values <0.05 were considered as significant. All statistical analyses were performed using the SAS statistical package version 9.3 (SAS Institute Inc., Cary, NC, USA). Statistical analyses were supported by the Institut für Herzinfarktforschung, Ludwigshafen, Germany. The authors had full access to and take full responsibility for the integrity of the data. All authors have read and agreed to the manuscript as written. The presented 30-day, 6-month, and one-year follow-up (FU) data are based on phone-call interviews.

## 4. Results

**Figure 4:**
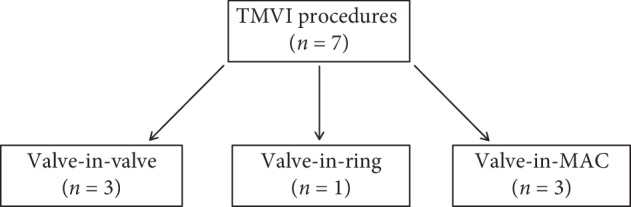
Flow chart of patients treated in this series according to the type of procedure (MAC = mitral annular calcification).

**Figure 5:**
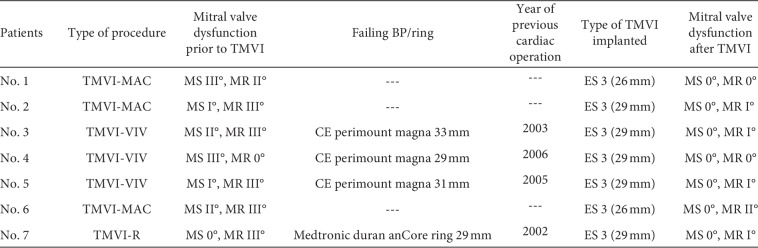
Detailed baseline and procedural characteristics of all patients treated by TMVI.

Between October 2016 and February 2018, seven patients (6 women and 1 man) underwent interventional mitral valve implantation in 7 procedures at our site. Patient demographics and comorbidities are displayed in Table 1. Three procedures were performed as valve-in-valve implantation (TMVI-VIV) in degenerated mitral bioprosthesis, three procedures were performed as valve-in-mitral-annular-calcification (TMVI-MAC) implantation, and one procedure was performed as valve-in-annuloplasty-ring implantation (TMVI-R) (Figure 4). Mean age of the patients treated was 77 ± 8.1 years, and 86% of them were female. Comorbidities are shown in Table 1. Two patients had prior surgical aortic valve replacement; one patient had received an aortic bioprosthesis, and the other patient had received a mechanical prosthesis. Mean ejection fraction of the population was 51 ± 13%. All patients were discussed in a Heart-Team-Conference prior to TMVI and were refused for conventional mitral valve reoperation for surgically high or prohibitive risk. Mean log EuroScore I was 39 ± 0.12%, reflecting a population at very high surgical risk. Mean time since last operation on the mitral valve was 161 ± 24 months. The primary dysfunction of the native mitral valve, the bioprosthesis, or the annuloplasty ring leading to TMVI was severe mitral regurgitation in 72% and severe mitral stenosis in 29% (Figure 5). No patient had significant paravalvular regurgitation after previous surgical mitral valve replacement prior to TMVI. The median number of previous thoracotomies was 1 (interquartile range (IQR), 1 to 1; one patient had two prior thoracotomies). Six out of seven patients had at least one previous surgical mitral or aortic valve replacement or surgical mitral valve repair using an annuloplasty ring. Indication for TMVI was the presence of severe heart failure symptoms (NYHA class III/IV, *n* = 7) in every patient (Table 1).

Procedural characteristics are shown in Table 2. A transseptal access was used in every patient (100%) treated in this series. Median procedural duration was 60 min (IQR, 60 to 83 min). Median fluoroscopy time was 11 min (IQR, 10 to 15 min), and median area dose product was 4934 cGy ∗ cm^2^ (IQR, 3349 to 8417 cGy ∗ cm^2^) (Table 2). In all procedures, no contrast was used during TMVI. Each patient was treated by implantation of a single Edwards SAPIEN 3 prosthesis. There was no periprocedural death (within 24 hours after procedure) in this series of patients treated by TMVI. All patients were extubated in the hybrid operating room or shortly afterwards on the intensive care unit.

### 4.1. In-Hospital Clinical Outcome

Complete technical procedural success could be achieved in 100%, and each patient underwent TMVI in one procedure. Clinical success was also achieved in 100% of patients with an improvement of at least one NYHA functional class after TMVI compared with preprocedure. Median NYHA class of 4 (IQR, 3 to 4) prior to procedure decreased to 2 (IQR, 2 to 2.5) after TMVI. This rate of improvement in NYHA class after TMVI was significant (*p*=0.008). Two patients improved by two functional NYHA classes, and five patients improved by one functional NYHA class. In echocardiographic assessment using continuous-wave Doppler, pmean was measured <5 mmHg after TMVI in every patient, which indicates no prosthetic stenosis due to a patient-prosthesis mismatch of the implanted Edwards SAPIEN 3 prosthesis. One patient had moderate paravalvular mitral valve regurgitation after TMVI-MAC, without a need for interventional paravalvular closure by plug implantation. All other patients showed no or mild mitral valve regurgitation after TMVI. Mean time from procedure to hospital discharge was 12 ± 6.1 days. In-hospital clinical outcome is shown in Table 3.

One patient died 12 days after procedure within the in-hospital period due to severe hospital-acquired pneumonia and sepsis with multiorgan failure after technically successful TMVI and initial clinical stabilization during the first days after TMVI. In this patient, alternating left-right cardiac shunt due to a large ASD was seen, and interventional closure was initially planned but could not be performed due to rapid clinical deterioration due to severe sepsis.

### 4.2. Complications

In two patients (one TMVI-VIV and one TMVI-MAC) with a persisting large ASD with clinically relevant interatrial shunt in TOE follow-up, interventional ASD closure was performed in a staged procedure using an Amplatzer Septal Occluder (Abbott, Illinois, USA). In a third patient, after TMVI-MAC, a relevant ASD with an alternating left-right cardiac shunt was seen, and interventional closure was planned. Unfortunately, this patient suffered from hospital-acquired pneumonia with severe sepsis and died 12 days after procedure. One patient treated by TMVI-VIV developed a complete atrioventricular block with a need for permanent pacemaker implantation afterwards. There was no access site bleeding complication, no LVOT obstruction by the implanted prosthesis, or no malpositioning or embolization of the prosthesis in this series of patients treated. No patient required mechanical circulatory support during or after TMVI.

### 4.3. Clinical Outcome at 30-Day, 6-Month, and One-Year Follow-Up (FU)

**Figure 6:**
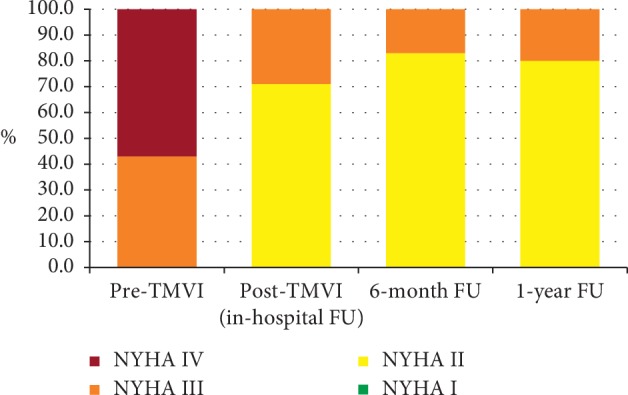
Comparison of NYHA functional classes before TMVI and at in-hospital, 6-month, and 1-year FU.

30-day, 6-month-, and 1-year follow-up of the population treated was complete in 100%. Clinical outcome is displayed in Table 3. After hospital discharge, no death occurred during one-year FU. Survival rates for patients were 86% (*n* = 6/7) at 30-day and 6-month FU. At one-year FU after TMVI, survival was 83% (*n* = 5/6). Clinical improvement according to NYHA functional class remained stable during one-year follow-up. Median NYHA class decreased to 2 (IQR, 2 to 2) at 30-day FU and remained stable during one-year after TMVI (*n* = 5; *p*=0.031).  Figure 6 shows the development in NYHA functional classes before TMVI and at in-hospital-, 6-month-, and 1-year FU.

## 5. Discussion

This limited series of patients treated by percutaneous transseptal TMVI at a single interventional center using Edwards Sapien 3 prostheses show encouraging results with a reasonable short- and midterm clinical efficacy at an acceptable procedural safety.

TMVI using an aortic transcatheter heart valve in the mitral position has been reported for the first time in 2010 by Webb et al. as a “valve-in-valve” procedure (TMVI-VIV) via a transseptal access [23]. Due to initial complications and difficulties with coaxial alignment of the transcatheter heart valve in the mitral bioprosthesis via a transseptal access, most following TMVI procedures were performed using a transapical access. In the recent years, TMVI has been performed predominantly as “valve-in-valve” or “valve-in-ring” procedures. Consecutively, there is some limited evidence on safety and efficacy on both procedures consisting of case series or smaller clinical registries to date [5, 7, 24–26]. Nevertheless, no randomized clinical trial on TMVI is available so far comparing interventional and surgical treatment options. Recently, TMVI as “valve-in-native-mitral-annular-calcification” (TMVI-MAC) has successfully been performed in patients who were either at very high surgical risk or inoperable. However, besides the proof of technical feasibility, data on TMVI-MAC are still very limited to date and consists of case reports and two smaller clinical registries only [12, 13, 15, 27–29].

A clinical and procedural success rate of 100% in the underlying small population treated by TMVI at our site starting its program seems encouraging in relation to the complexity of those interventions performed in a patient cohort at an increased risk for repeat surgery. All patients in the underlying small series treated by TMVI showed symptomatic improvement during the first days after procedure. High procedural and clinical success rates are in accordance with previous reports from clinical registries after TMVI-VIV or TMVI-R, ranging from 82% to 100% regarding procedural success and from 92% to 95% regarding clinical success [24, 26, 30–32]. TMVI-MAC is technically even more challenging and postprocedural results are less predictive compared with TMVI-VIV and TMVI-R. Recently, first results after TMVI-MAC showed a procedural success rate between 67% and 84% and a clinical success rate in approximately 92% of patients at 30-day follow-up [28, 29, 31].

In-hospital and 30-day mortality was 14% in the underlying small series of patients treated by transseptal TMVI, which is also in accordance to another reported 30-day mortality rate of 15% by Eleid et al. after TMVI performed for all three different indications (TMVI-VIV, TMVI-R, and TMVI-MAC) [31]. One-year mortality was 17% in the underlying population after transseptal TMVI. Other reports have shown one-year mortality rates ranging from 9% to 22% after TMVI-VIV or TMVI-R [24, 33].

As previously mentioned, patients undergoing TMVI-MAC are a special subpopulation of patients at generally high morbidity and mortality rates. In this context, the only available multicenter registry on TMVI-MAC recently reported a 30-day mortality rate of 30.7% in the subgroup of patients treated by transseptal TMVI-MAC, which is in accordance to the underlying 30-day mortality rate after TMVI-MAC of 33% (*n* = 1/3, TMVI-MAC).

The majority of patients in this cohort treated by TMVI were female (86%). This finding is not unusual and was also approved by different reports on TMVI, which showed higher rates of women undergoing TMVI in clinical practice compared with men (ranging from 60% to 77%) [24, 28, 29, 33].

Transseptal TMVI is a complex and often technically demanding procedure for the echocardiologist, and the operations are performed in a population at an increased risk for morbidity and mortality. It has been shown that transseptal TMVI-VIV can be performed at relatively low procedural risk with high procedural success rates [7, 8, 24–26, 30, 31, 33]. TMVI-R seems to be more technically challenging procedures due to some uncertainties regarding preprocedural planning and prediction of postprocedural technical results with respect to dimensions, shapes, and contours between rings and bioprosthesis. In this context, a very exact positioning of an adequately sized transcatheter heart valve into a complete annuloplasty ring seems of paramount importance to prevent significant paraprosthetic regurgitation or LVOT obstruction afterwards. Furthermore, a stable anchoring of the prosthesis in a complete mitral annuloplasty ring seems crucial to prevent migration of the prosthesis afterwards, which has been described as one possible complication after TMVI-R [31]. TMVI-R has been associated with higher rates of paravalvular regurgitation and mortality compared with patients undergoing TMVI-VIV [34]. In the only patient treated by TMVI-R in this series, only trace paravalvular regurgitation and no LVOT obstruction occurred. However, we report on a very limited series of patients treated (*n* = 7), which make a comparison of relative complication and mortality rates to larger registry data impossible.

TMVI-MAC is the most challenging procedure, and postprocedural results are reported to be worse and sometimes hard to predict compared with TMVI-VIV/TMVI-R at this time. In our series of patients treated, all three procedures were technically successful. Nevertheless, one of three patients showed a moderate paravalvular mitral regurgitation and a large, persisting, clinically relevant ASD after TMVI-MAC. Unfortunately, this patient died during the in-hospital course due to severe pneumonia and sepsis, and we cannot exclude an association of the large ASD or the moderate paravalvular regurgitation after procedure with the in-hospital death. In this context, reports on TMVI-MAC have shown increased complication rates, including valve embolization, LVOT obstruction, or the need to conversion to open heart surgery compared with TMVI-VIV/TMVI-R [28]. However, with respect to the complexity of the native mitral valve anatomy (saddle-oval shape, interaction with the LVOT and the aortic valve) treated by a balloon-expandable, circular-shaped transcatheter heart valve, which was not developed to treat the mitral valve, procedural and clinical results seem promising. Nevertheless, further studies are needed to standardize preprocedural planning and procedural aspects to further improve postprocedural results. In this context, many questions need to be answered in the future, like the best method for mitral annulus sizing, the amount of calcium burden needed to securely anchor the prosthesis in the calcified native mitral ring, or the optimal height of implantation in relation to the mitral annular plane to prevent LVOT obstruction or paravalvular regurgitation. Therefore, especially TMVI-MAC should presently be performed at specialized sites in patients without surgical treatment options, who are enrolled in clinical registries only.

## 6. Conclusions

Transvenous TMVI-VIV and TMVI-R can be performed at high efficacy and procedural safety and are reasonable treatment options in patients with failing mitral bioprosthesis or annuloplasty rings at high surgical risk, today. Therefore, TMVI-VIV and TMVI-R have the ability to become a first-line approach in the treatment of patients at higher surgical risk suffering from failing mitral valve bioprosthesis or annuloplasty rings in the future. At this time, TMVI-MAC is feasible but is still associated with a higher complication rate and worse procedural results compared with TMVI-VIV/TMVI-R. Nevertheless, in-hospital and midterm clinical outcome seems encouraging, and TMVI-MAC might be a reasonable treatment option in carefully selected patients. For high procedural demands, TMVI need to be performed by experienced operators at specialized sites to achieve best clinical results. Every patient treated by TMVI-VIV, TMVI-R, or TMVI-MAC should be enrolled into a large clinical registry to gain more information especially on longer-term safety and efficacy of this relatively new therapeutic approach.

## 7. Limitations

The number of patients and procedures in this series is small, and women were predominantly treated by TMVI in this series of patients. Therefore, the results cannot be generalized to other populations undergoing TMVI. However, our results are in accordance with other reports on TMVI in similar populations. Efficacy and safety of TMVI in the underlying series seems encouraging regarding the complexity of the procedures. Clinical FU was complete in 100% at 30 days, 6 months, and one year and was performed by telephone interviews. One of seven patients treated by TMVI in 2018 did not reach one-year FU until this article was prepared. Therefore, we can report the one-year FU of six patients, only. Clinical FU included information on survival and symptomatic status (NYHA functional class), only. Routine echocardiographic analyses were not part of the FU after hospital discharge. Therefore, we cannot report on residual valve regurgitation and technical long-term success after hospital discharge. However, persistent clinical improvement in the majority of patients during 1-year FU strongly suggests stable technical results, also. Results from smaller series need to be confirmed by a large multicenter TMVI registry.

## Figures and Tables

**Table 1:** Patient demographics and comorbidities.

Patients treated (*n* = 7)	
Female gender	86%

Age (years, mean, SD)	77 ± 8.1

Time since last mitral valve operation (month, mean, SD, *n* = 4)	161 ± 24

Number of thoracotomies per patient (median, IQR)	1 (1 to 1)

NYHA functional class prior to procedure	
NYHA class IV	57% (4/7)
NYHA class III	43% (3/7)
NYHA class II	0% (0/7)
NYHA class I	0% (0/7)

Leading mitral valve dysfunction (native mitral valve, annuloplasty ring, or prosthesis)	
Severe mitral valve regurgitation, ≥grade III	71% (5/7)
Severe mitral valve stenosis, ≥grade III	29% (2/7)

Prior aortic valve replacement	
TAVR	0% (0/7)
SAVR	29% (2/7)
Bioprosthetic	14% (1/7)
Mechanical	14% (1/7)

Comorbidities	
Chronic renal impairment (creatinine > 1.5 mg/dl)	57% (4/7)
Coronary artery disease	57% (4/7)
Prior CABG	43% (3/7)
Severe pulmonary hypertension (PAsys > 50 mmHg)	100% (7/7)
Atrial fibrillation	86% (6/7)
Prior stroke	29% (2/7)
Prior permanent pacemaker or ICD implantation	43% (3/7)
Chronic lung disease	71% (5/7)

Ejection fraction (mean)	51 ± 13%

Log. EuroSCORE I (mean)	39%

**Table 2:** Procedural characteristics.

Number of procedures (total)	7
Access for TMVI	
Transseptal/transvenous	100% (7/7)
Transapical	0% (0/7)
Transatrial	0% (0/7)
Complete technical procedural success (according to MVARC)	100% (7/7)
Periprocedural death (within 24 hours)	0% (0/7)
Procedural duration (minutes, median, IQR)	60 (60; 83)
Fluoroscopy time (minutes, median, IQR)	11 (10; 15)
Area dose product (cGy ∗ cm^2^, median, IQR)	4934 (3349; 8417)

**Table 3:** Clinical outcome (in-hospital, 30-day-, 6-month- and one-year follow-up).

Patients treated: *n* = 7	
In-hospital follow-up	
Clinical success (with improvement in at least one NYHA functional class after procedure)	100% (7/7)
NYHA functional class post procedure	
Improvement by two NYHA functional classes	29% (2/7)
Improvement by one NYHA functional class	71% (5/7)
No improvement in NYHA functional class	0% (0/7)
NYHA functional class after procedure	
NYHA class IV	0% (0/7)
NYHA class III	29% (2/7)
NYHA class II	71% (5/7)
NYHA class I	0% (0/7)
Residual mitral regurgitation after TMVI at hospital discharge	
None	29% (2/7)
Trace or mild (MR grade I)	57% (4/7)
Moderate (MR grade 2)	14% (1/7)
Severe (MR grade 3)	0% (0/7)
Complications	
Vascular access site bleeding complication	0% (0/7)
Device embolization	0% (0/7)
Need for second valve implantation	0% (0/7)
Cardiac perforation/cardiac tamponade	0% (0/7)
Major stroke	0% (0/7)
New arrhythmia	14% (1/7)
Conversion to open heart surgery	0% (0/7)
Acute kidney injury	0% (0/7)
LVOT obstruction by implanted mitral valve prosthesis	0% (0/7)
Major atrial septal defect (ASD) after TMVI, hemodynamically relevant	43% (3/7)
Interventional ASD closure	29% (2/7)
Pacemaker implantation post TMVI	14% (1/7)
In-hospital mortality rate	14% (1/7)
In-hospital stay from TMVI to hospital discharge (days, mean ± SD)	12 ± 6.1
Clinical follow-up after hospital discharge	
30-day mortality rate (all-cause)	14% (1/7)
6-month mortality rate (all-cause)	14% (1/7)
One-year mortality rate (all-cause, *n* = 6)	17% (1/6)
NYHA functional class at 30-day and 6-month follow-up (*n* = 6)	
NYHA class IV	0% (0/6)
NYHA class III	17% (1/6)
NYHA class II	83% (5/6)
NYHA class I	0% (0/6)
NYHA functional class at 1-year follow-up (available in *n* = 5)	
NYHA class IV	0% (0/5)
NYHA class III	20% (1/5)
NYHA class II	80% (4/5)
NYHA class I	0% (0/5)

## Data Availability

The data used to support the findings of this study are available from the corresponding author upon request.
